# Evolutionary Genomics Implies a Specific Function of *Ant4* in Mammalian and Anole Lizard Male Germ Cells

**DOI:** 10.1371/journal.pone.0023122

**Published:** 2011-08-12

**Authors:** Chae Ho Lim, Takashi Hamazaki, Edward L. Braun, Juli Wade, Naohiro Terada

**Affiliations:** 1 Department of Pathology, College of Medicine, University of Florida, Gainesville, Florida, United States of America; 2 Department of Biology, College of Liberal Arts and Sciences, University of Florida, Gainesville, Florida, United States of America; 3 Neuroscience Program, Department of Psychology, Department of Zoology, Michigan State University, East Lansing, Michigan, United States of America; Oxford Brookes University, United Kingdom

## Abstract

Most vertebrates have three paralogous genes with identical intron-exon structures and a high degree of sequence identity that encode mitochondrial adenine nucleotide translocase (*Ant*) proteins, *Ant1* (*Slc25a4*), *Ant2* (*Slc25a5*) and *Ant3* (*Slc25a6*). Recently, we and others identified a fourth mammalian *Ant* paralog, *Ant4* (*Slc25a31*), with a distinct intron-exon structure and a lower degree of sequence identity. *Ant4* was expressed selectively in testis and sperm in adult mammals and was indeed essential for mouse spermatogenesis, but it was absent in birds, fish and frogs. Since *Ant2* is X-linked in mammalian genomes, we hypothesized that the autosomal *Ant4* gene may compensate for the loss of *Ant2* gene expression during male meiosis in mammals. Here we report that the *Ant4* ortholog is conserved in green anole lizard (*Anolis carolinensis*) and demonstrate that it is expressed in the anole testis. Further, a degenerate DNA fragment of putative *Ant4* gene was identified in syntenic regions of avian genomes, indicating that *Ant4* was present in the common amniote ancestor. Phylogenetic analyses suggest an even more ancient origin of the *Ant4* gene. Although anole lizards are presumed male (XY) heterogametic, like mammals, copy numbers of the *Ant2* as well as its neighboring gene were similar between male and female anole genomes, indicating that the anole *Ant2* gene is either autosomal or located in the pseudoautosomal region of the sex chromosomes, in contrast to the case to mammals. These results imply the conservation of *Ant4* is not likely simply driven by the sex chromosomal localization of the *Ant2* gene and its subsequent inactivation during male meiosis. Taken together with the fact that Ant4 protein has a uniquely conserved structure when compared to other somatic Ant1, 2 and 3, there may be a specific advantage for mammals and lizards to express *Ant4* in their male germ cells.

## Introduction

The adenine nucleotide translocase (Ant), also called ADP/ATP carrier (Aac), mediates the exchange of ADP and ATP across the inner mitochondrial membrane, thus playing an essential role in energy metabolism in eukaryotic cells [1]–[3]. Under respiring conditions, ATP produced within the mitochondria is exported to the cytosol through Ant to support cellular activities. In exchange, ADP is imported to provide a substrate for the conversion of ADP to ATP by ATP synthase. Ant belongs to the mitochondrial carrier family that supports a variety of transport activities across the mitochondrial inner membrane [3], [4]. A typical Ant molecule comprises 300–320 amino acid residues that form six transmembrane helices.

The Ant family proteins are encoded by the nuclear genome. As free living bacteria lack Ant-like molecules, Ant proteins are thought to have evolved from a broad specificity transport family of eukaryotic origin [5]. Most eukaryotes, including unicellular eukaryotes, have multiple Ants. For example, the budding yeast *Saccharomyces cerevisiae* has three genes (*AAC1*, *AAC2*, and *AAC3*) that encode Ant proteins [6]. Among them, Aac2p is a major isoform that is abundantly expressed during respiration and repressed during fermentation [7]. Aac1p and Aac3p are expressed almost exclusively in aerobic and anaerobic conditions, respectively [6], [8]. Moreover, Aac2p and Aac3p have an ability to import ATP that is sufficient for survival of yeast after loss of the mitochondrial genome whereas Aac1p does not [9]. Thus, different Ants are likely utilized for ATP export and import, in order to efficiently cope with varying external nutrient and oxygen conditions. Interestingly, the *S. cerevisiae AAC1* gene appears to have accumulated substitutions at a higher rate that *AAC2/3* [10], consistent with the idea that the *AAC1* gene underwent a functional change after duplication. Phylogenetic analysis has the ability to provide insight into the functions of the *Ant* genes in vertebrates.

In multicellular organisms, differential expression of *Ant* genes depends on a variety of factorsincluding tissue type, developmental stage, and cellular proliferation state. Most vertebrates possess three distinct *Ant* paralogs that exhibit a relatively high degree of sequence identity. Of these, *Ant1* (*Slc25a4*) is expressed primarily in the heart and skeletal muscle, and is presumed to be suitable for rapid ATP metabolism in heart and skeletal muscles [11]. Genetic inactivation of *Ant1* (*Ant1*−/−) resulted in viable mice; however, these animals developed mitochondrial myopathy and severe exercise intolerance along with hypertrophic cardiomyopathy as young adults [12]. *Ant2* (*Slc25a5*) and *Ant3* (*Slc25a6*) are expressed ubiquitously in somatic tissues; however, *Ant2* expression is higher in rapidly growing cells and is inducible in mammals whereas *Ant3* appears to be constitutively expressed in all tissues [13], [14]. It should be noted that rodents lack the *Ant3* ortholog; instead, the mouse *Ant2* ortholog likely carries out the functions of human *Ant2* and *Ant3* [15], [16].

Utilizing various approaches, we and others identified a fourth member of the *Ant* gene family, *Ant4* (also called *Slc25a31*, *Aac4*, and *SFEC*) in both humans and mice [17]–[19]. The human *ANT4* gene was predicted to encode a 315 amino acid protein with a relatively high degree of amino acid sequence identity to the previously identified ANT proteins (73%, 71% and 72% overall amino acid identity to ANT1, ANT2 and ANT3, respectively). Like all other *ANT* genes, *ANT4* encodes a protein that has three tandem repeats of an approximately 100 amino acids containing two transmembrane regions, which is a characteristic shared by all members of the solute carrier family [20]. Further, the Ant4 protein contains a RRRMMM sequence at the end of the fifth α-helix transmembrane domain, a sequences that is conserved in all of the Ant proteins (but not in other Slc25s) that is essential for ADP/ATP transport activity [21]. Indeed, Dolce et al. [18] reported that Ant4 specifically exchanges ADP and ATP, but not other solutes, by an electrogenic antiport mechanism.

We have determined that *Ant4* is expressed exclusively in testicular germ cells in adult mice, with its expression being particularly high during meiosis [22]. It should be noted here that *Ant4* appears to be expressed in embryonic ovaries as well [23](Lim et al., unpublished observation). Further, *Ant4* is essential for male germ cell development in mice, as *Ant4* null male mice are infertile and exhibit meiotic arrest around the leptotene stage [22], [24]. The *Ant2* gene is always located on the X chromosome of mammals; thus, *Ant2* gene expression is repressed during male germ cell meiosis due to meiotic sex chromosome inactivation (MSCI) [22]. On the other hand, the *Ant4* gene is always located on an autosome. A plausible hypothesis regarding the primary function of *Ant4* is that it acts to compensate for the loss of *Ant2* expression during male meiosis in mammals [22]. The meiotic arrest phenotype in *Ant4* null male mice is indeed consistent with this compensation theory [24], and this theory would be consistent with the presence of *Ant4* only in mammals. Since *Ant4* is present in both eutherian and metatherian mammals, this hypothesis would place the duplication sometime between 150 million years ago (MYA), when the eutherian-metatherian divergence occurred [25], [26], and 300 MYA, when synapsids (Synapsida, the clade that includes mammals) diverged from the birds and true reptiles (clade Reptilia) [27].

This hypothesis is indirectly challenged by phylogenetic analyses that suggested a more ancient origin of *Ant4* [22]. Here we present direct evidence for a more ancient origin of *Ant4*. Briefly, the anole lizard has an ortholog of *Ant4* that exhibits testicular expression similar to that seen in mammals; however the anole *Ant2* gene was not located on a sex chromosome. Moreover, additional phylogenetic analyses confirm that the *Ant* gene family in animals has a complex history of gene duplications and losses and further indicate that the *Ant4* subgroup likely has an ancient origin. Based on these findings, we describe our new hypothesis, which is not necessarily mutually exclusive with the compensation theory, that there is a specific advantage for mammals and lizards to express *Ant4* during spermatogenesis and/or in their sperm.

## Results

### Anole lizard has the *Ant4* ortholog

**Figure 1:**
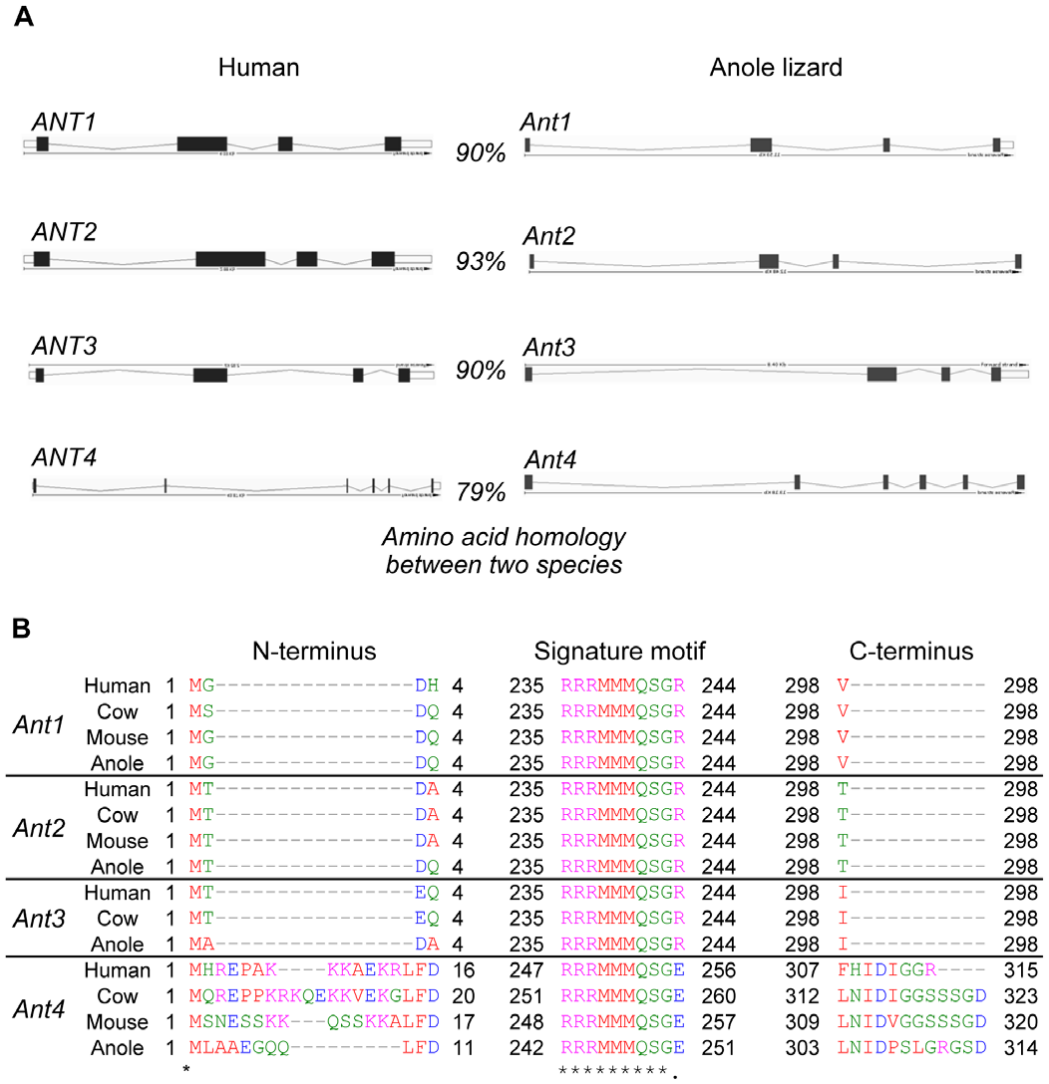
Anole lizard has orthologs of the *Ant1*, *2*, *3*, and *4* genes. (**A**) The schematic diagram from the Ensemble database shows the configuration of genes encoding Ant proteins in human and anole lizard. Exons and introns are shown as black boxes and lines, respectively. Numbers represent the amount of amino acid sequence identity that the products of each *Ant* gene in the human and anole lizard exhibit. (**B**) Alignment of the inferred amino acid sequences of the products of the *Ant1*, *2*, *3*, and *4* genes in mammals (human, cow, mouse) and reptile (anole lizard). Ant amino acid sequences of the selected species were aligned using ClustalW2. Amino acids sequence surrounding a signature motif in the Ant4 protein (RRRMMM) and the N- and C-terminal parts of the proteins are shown. Amino acid residues are numbered from the initiation codon (M) to the termination codon in each Ant protein.

The green anole lizard, *Anolis carolinensis*, is the first non-avian reptile genome to be sequenced. When we examined conservation of *Ant* gene orthologs in the anole, we found likely orthologs of all four *Ant1*, *2*, *3* and *4* genes present in humans. Anole *Ant1, 2, 3 and 4* genes encode proteins with 90%, 93%, 90% and 79% identity to human orthologs, respectively. Moreover, their intron-exon structure was conserved between the anole and mammals, with four exons observed in *Ant1*, *2* and *3* and six exons in *Ant4* (**Fig. 1A**). Indeed, the deduced amino acid sequence of anole Ant4 had unique features only seen in the mammalian Ant4, including N-terminal and C-terminal extensions and a RRRMMMQSGE amino acid sequence at the end of the helix H5 region (**Fig. 1B**). To the best of our knowledge, this was the first time a clear ortholog of *Ant4* has been identified outside of mammals.

**Figure 2:**
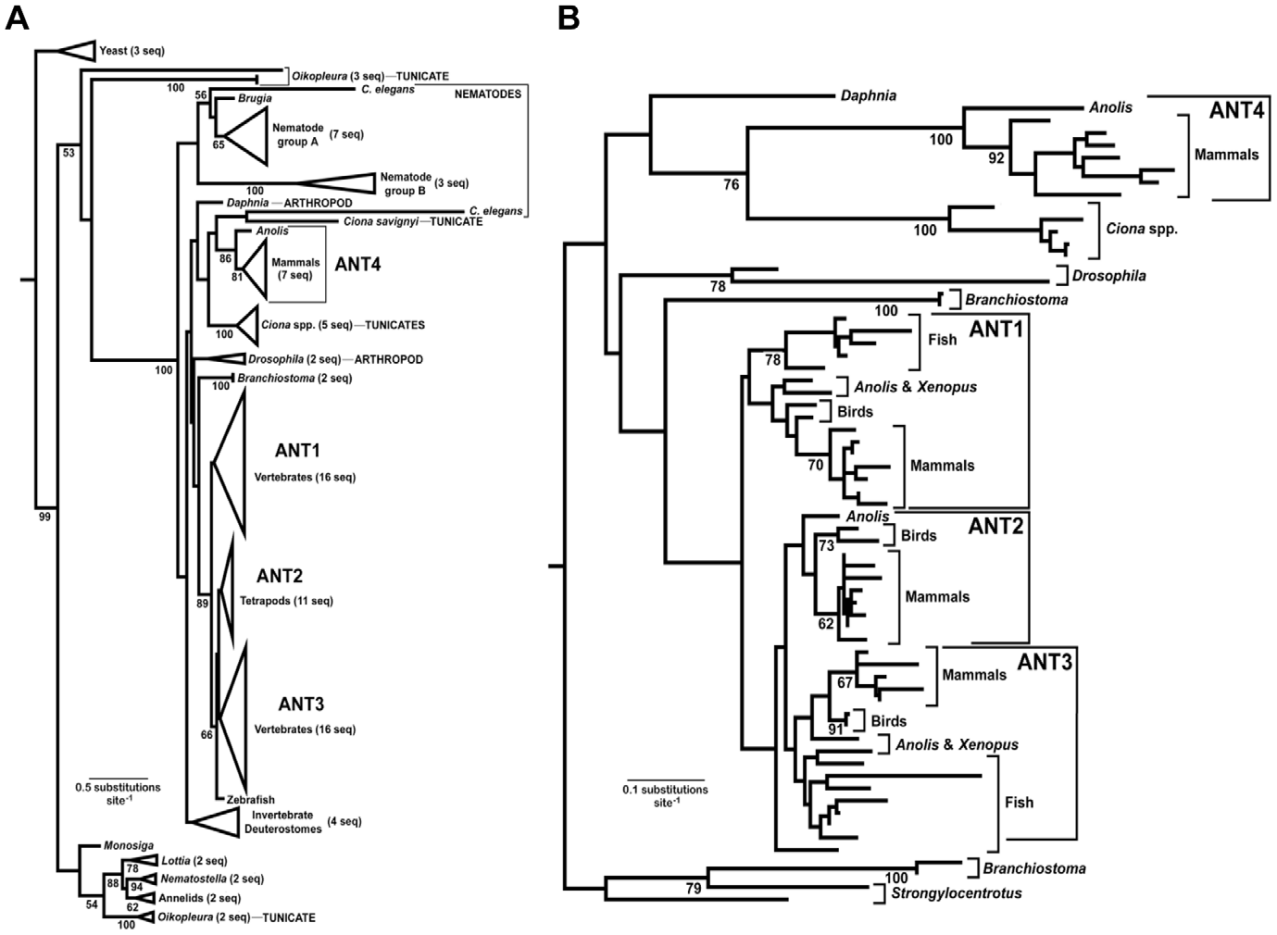
The maximum-likelihood (ML) estimate of *Ant* phylogeny supports an ancient origin of *Ant4*. (**A**) Large scale phylogeny of animal *Ant* homologs obtained by ML analysis of amino acid sequences using the LG+Γ+F model of evolution. Support based upon 500 bootstrap replicates is shown as a percentage adjacent to the relevant branches when it exceeds 50%. This phylogeny included a fungal (*Saccharomyces cerevisiae*) outgroup, and the root of the tree was assumed to lie between fungi and the animal-choanoflagellate clade (the latter group is represented by *Monosiga brevicollis*). This phylogeny suggests a number of ancient gene duplications, one of which led to a “core group” that includes the vertebrate *Ant* homologs. (**B**) Phylogeney of the “core group” obtained using the same model. Support based upon 500 bootstrap replicates is shown as a percentage adjacent to the relevant branches when it exceeds 50%; support for some clades within well-supported groups (e.g. branches within teleost fish) was omitted in the interest of simplicity. Analyses using this more limited taxon sample were conducted to test the possibility that the position of *Ant4* was influenced by the inclusion of divergent sequences. The position of *Ant4* was robust to the taxon set analyzed; in fact, the bootstrap support for an ancient origin (the *Ant4*-tunicate clade) increased for the smaller taxon sample.

Consistent with the intron-exon structure and the signature sequences, phylogenetic analyses (**Fig. 2A**) verified the relationship between anole and mammalian *Ant4* and further suggested that the *Ant4* lineage is more closely related to the specific invertebrate *Ant* genes than it is to *Ant1*, *2*, or *3*. These analyses suggest the occurrence of a large number of gene duplications (and gene losses, given the inclusion of many organisms with relatively complete genome sequences that were included in our analysis) and further indicate that vertebrate *Ant* genes are found in a “core group” that also includes some invertebrate sequences. This core group was united by a long branch with a high degree of bootstrap support. Within this group, however, bootstrap support was relatively limited. Since the inclusion of very divergent outgroup sequences has the potential to distort the ingroup topology [29], [30] we excluded the outgroup sequences (along with two very divergent ingroup sequences) and repeated the phylogenetic analysis (**Fig. 2B**). These analyses confirmed the relationship between a set of tunicate *Ant4*-like genes and vertebrate Ant4 orthologs. Although the relevant tunicate genes are intronless and lack the signature sequences of vertebrate *Ant4* orthologs, the phylogenetic analyses support the hypothesis that the divergence between the *Ant4* group and the *Ant1*-*3* group reflects an ancient duplication, substantially predating the origin of vertebrates.

### Anole *Ant4* is expressed in testis

**Figure 3:**
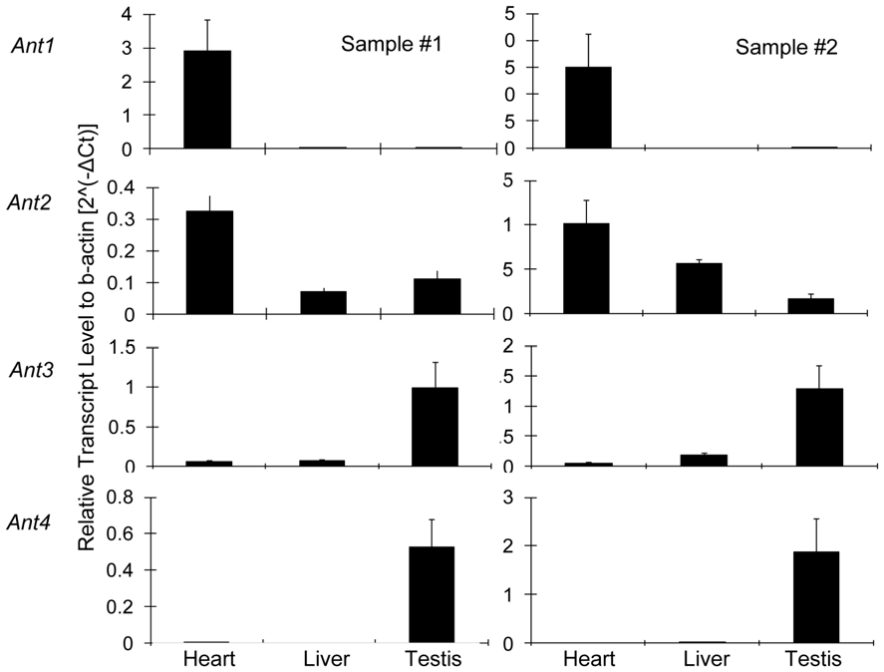
Anole *Ant4* is expressed in testis. *Ant1*, *2*, *3* and *4* gene expression in heart, liver, and testis of anole lizard was examined by qRT-PCR. *Ant* mRNA amounts were normalized based upon the expression of the *β-actin* mRNA. Error bars indicate standard deviations of triplicate samples.

We next examined the gene expression pattern of *Ant1*, *2*, *3* and *4* in anole lizard. Heart, liver and testis were harvested from male anole lizard, and total RNA was extracted. The amount of *Ant* mRNA in these organs was then examined using quantitative RT-PCR (**Fig. 3**). *Ant1*, which is selectively expressed in heart and skeletal muscle in mammals, was also expressed in anole heart but was not detectable in anole liver and testis. *Ant2* and *Ant3*, which are expressed ubiquitously in mammals, were also expressed in all of the anole tissues examined. *Ant4*, which is expressed exclusively in testis in adult mammals, was also expressed in anole testis but not in any somatic organs examined. These data indicated that all *Ant* orthologs exhibit similar patterns of gene expression in the anole and mammals. In particular, testicular expression of *Ant4* suggests this gene likely plays a role in anole male germ cells, similar to the role of *Ant4* in mice.

### Chicken has a degenerate DNA fragment that corresponds to a likely *Ant4* pseudogene

**Figure 4:**
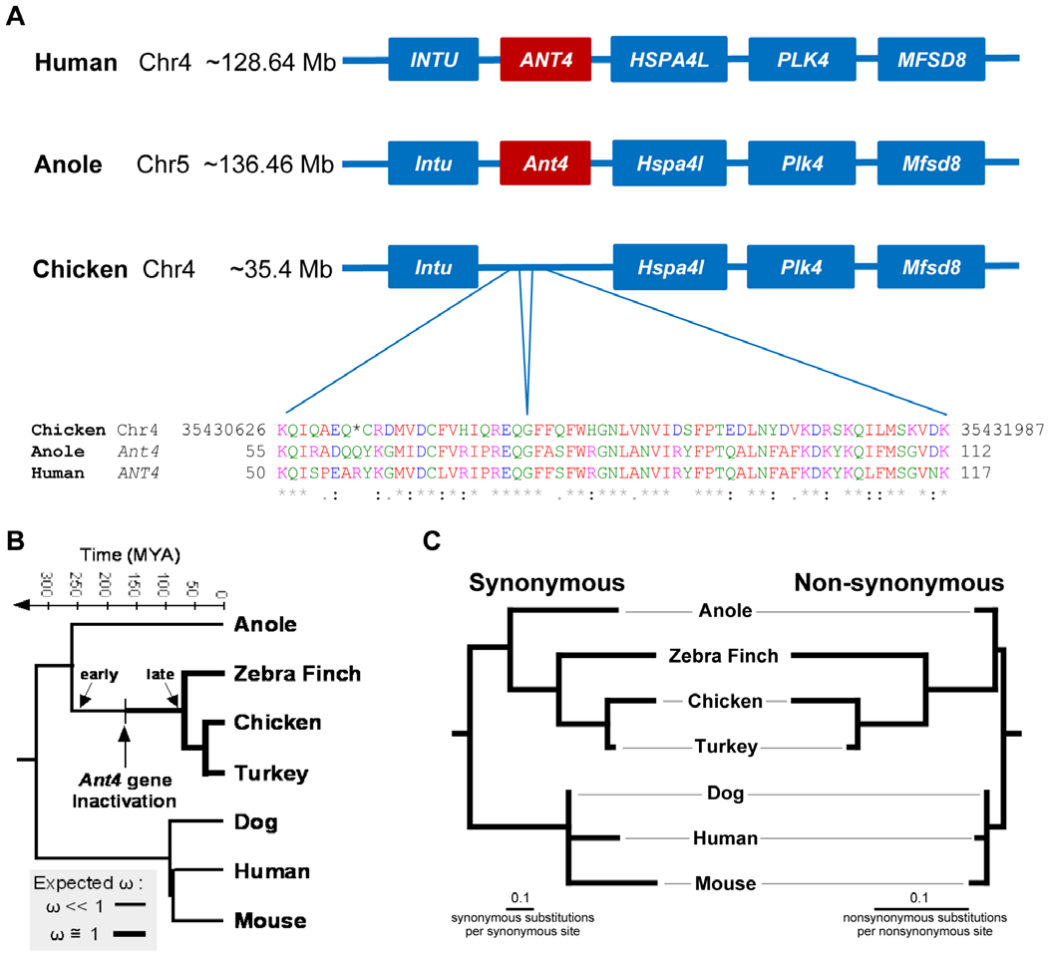
Degenerate DNA fragment of putative *Ant4* gene in the syntenic region of chicken genome. (**A**) The syntenic region that includes *Ant4* in the human, anole lizard and chicken genomes. The schematic diagram shows *Ant4* (red boxes) with chromosomal location flanked by neighboring genes (blue boxes). Translated amino acid sequence of degenerate DNA regions of the putative *Ant4* gene loci in the chicken genome was aligned with amino acid sequence of *Ant4* of human and anole lizard. The asterisk in the chicken sequence is a stop codon; additional inactivating mutations include a 2-bp frameshift between nucleotide that would encode the D and K at the end of the chicken sequence and a stop codon immediately after the K. (**B**) Vertebrate phylogeny showing approximate divergence times (in millions of years before present) and the expected changes in ω (see text for definition) for the early and late inactivation models. (**C**) Phylogeny with branch lengths reflecting numbers of synonymous and non-synonymous substitutions. The light gray lines are included to make it easier to identify the taxon associated with each terminal, they have no biological significance.

Based on phylogeny and intron-exon structure, the anole *Ant4* (*Slc25a31*) gene appears to be an ortholog of mammalian Ant4 genes. Moreover, the gene expression profile suggests that its function in the anole is similar to its function in mammals. This implies that the common ancestor of mammalian and reptilian species had the *Ant4* gene. It's logical to ask what happened to the *Ant4* gene in birds. To answer this question, we examined chromosomal regions harboring *Ant4* in other vertebrates. As shown in **Fig. 4A**, the human *ANT4* gene is located on chromosome 4q28.1, between the *INTU* gene and the *HSPA4L*, *PLK4*, and *MFSD8* genes. Chromosomal synteny is conserved between human and anole genomes and these genes flank the 5′- and 3′-end of the *Ant4* gene in the anole as well. These data further confirm that the anole *Ant4* gene is an authentic ortholog of the mammalian *Ant4* genes.

Examining the chicken genome demonstrated the syntenic region including *Intu*, *Hspa4l*, *Plk4*, *Mfsd8* genes were present in this order on chromosome 4, although an *Ant4* ortholog was absent from this region (**Fig. 4A**). However, it was possible to identify DNA fragments similar to the *Ant4* gene between *Intu* and *Hspa4l* genes in the chicken genome and other avian genomes. For example, the sequence of chromosome 4 in the chicken genome (nucleotides 35430626 through 35431987 in build WUGSC 2.1/galGal3) includes two genomic regions which could be translated to 23 and 40 amino acid fragments with a high degree of similarity to human and anole Ant4 proteins. However, there is one nonsense codon and a two bp insertion that creates a frameshift in these regions. Similar degenerate *Ant4*-like DNA sequences were also evident in the turkey and zebra finch genomes, although the zebra finch sequence only includes a region similar to the second of these exons. An intron-like sequence was present at a site identical to the first intron of the human and anole *Ant4* genes in the avian pseudo-coding regions, and these regions retain the GT sequence after the splice donor site and the AG before the splice acceptor (only the AG is retained in the zebra finch since the first exon-like region appears to have been deleted in this taxon). Moreover, the second exon is followed by a GT sequence in the zebra finch (this sequence is AT in the chicken and turkey, suggesting a mutation occurred in these lineages after their divergence from the finch lineage). These data confirm that the common ancestor of reptilian and mammalian species had the *Ant4* gene, as suggested by phylogenetic analyses (**Fig. 2**), and indicate that the *Ant4* gene has been degraded during avian evolution. Since we were not able to identify obvious synteny of the region in the zebrafish (*Danio rerio*) and frog (*Xenopus tropicalis*) genome, we were not able to elucidate whether degenerate *Ant4* pseudogenes are present outside the amniotes.

The evidence suggests the *Ant4* gene was inactivated in the common ancestor of the zebra finch (a member of the Passeriformes) and the chicken and turkey (both members of Galliformes). The avian supergroup (Neognathae) that includes both of these orders is very diverse, and includes virtually all orders of birds [31]. Given this constraint, it remains possible that the *Ant4* was inactivated at any point before the divergence of these lineages (**Fig. 4B**). To place the timing of this inactivation in a framework that can be tested using phylogenetic methods, we simplified this to two models, an early inactivation model and a late inactivation model (indicated by the arrows in **Fig. 4B**). Fitting a set of models of codon evolution to the nucleotide sequence data for *Ant4*, including the two avian *Ant4* pseudogenes, allows us to test these simplified models. The fundamental prediction is that the ω parameter, which describes the relative rates of nonsynonymous and synonymous substitutions, will increase to a value of one upon gene inactivation. As a heuristic, we present a comparison of trees with branch lengths proportional to the amounts of nonsynonymous and synonymous change (**Fig. 4C**). As expected for pseudogenes, the synonymous branch lengths were similar for birds and other taxa whereas the nonsynonymous branch lengths were much longer in birds (**Fig. 4C**). The length of the branch leading to the birds in the nonsynonymous tree was also long, a finding more consistent with the early inactivation model.

We tested five different models of codon evolution using this *Ant4* data. All of these models involved shifts in the ω parameter, and the model set included one model with two different ω parameters that was more consistent with the early inactivation model and one that was more consistent with late inactivation. This allowed us to put the branch length heuristic in more rigorous framework. The best-fitting model based upon the AIC was the two-ω early inactivation model. However, the alternative model was not excluded from the 95% confidence set of models (Table S1). Nonetheless, the early inactivation model is better supported by the estimates of the ω parameter (Table S1) from other alternative models. Taken as a whole, the majority of the data suggest an early inactivation.

### Anole *Ant2* is not on a heterogametic sex chromosome

**Figure 5:**
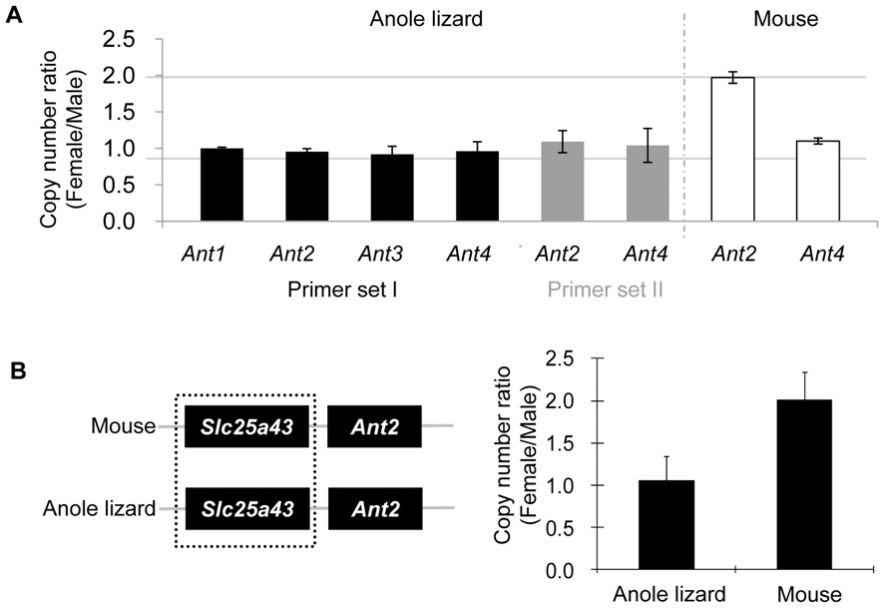
Gene dosage analysis in female and male animals. Relative copy number ratio of *Ant1*, *2*, *3* and *4* genes and *Slc25a43* gene in male and female animals were examined by quantitative PCR analysis of genomic DNA of anole lizard and mouse. PCR amplification was normalized to the control *β-actin* gene. Error bars indicate standard deviations of three independent experiments. (**A**) Relative gene dosages of the *Ant 1*, *2*, *3* and *4* between female and male in anole lizard and mouse. The result was confirmed with two different sets of primers for anole *Ant2* and *Ant4* genes. (**B**) Relative gene dosage of the *Slc25a43* between female and male in anole lizard and mouse. The *Slc25a43* gene localizes adjacent to the *Ant2* gene in both anole and mouse genome (left panel).

We have previously hypothesized that mammalian *Ant4* genes may be conserved to compensate for the loss of X-linked *Ant2* (*Slc25a5*) gene expression during male meiosis. It is important to note that anole lizards are considered male heterogametic reptiles, having XY chromosome differentiation [32], [33]. Thus, we hypothesized the anole *Ant2* gene might also be located on the X-chromosome. Since chromosomal localization data are not available for a number of green anole genes, here we determined the relative copy numbers of genes between male and female animals by quantitative genomic DNA PCR (**Fig. 5**). First, in order to verify the methods, we compared gene dosages of *Ant2* in male and female mice. When compared to the control *β-actin* gene, the relative copy number of the X-linked *Ant2* gene was approximately double in female mice when compared to male mice. In contrast, dosage of the autosomal *Ant4* gene was similar between male and female mice. These results indicate this is a valid method for determining relative copy number of genes. When we applied the method to anole lizard, relative copy number ratios were approximately one for all *Ant* genes, including *Ant2*. In addition, multiple primer sets were used to confirm the results for the *Ant2* and *Ant4* genes. Similarly, the relative copy number ratio between male and female were examined for *Slc25a43*, another gene encoding a solute carrier protein that is located adjacent to the *Ant2* gene in the syntenic region of both the mouse and anole genomes. As seen with the *Ant2* gene, the relative copy number ratio of the *Slc25a43* gene was approximately two in female and male mice but approximately one in female and male anole (**Fig. 5B**). Taken together, these data indicate that the anole *Ant2* gene is not located on a heterologous region of the X chromosome, but either on an autosome or on the pseudoautosomal regions of the anole X and Y chromosomes.

**Figure 6:**
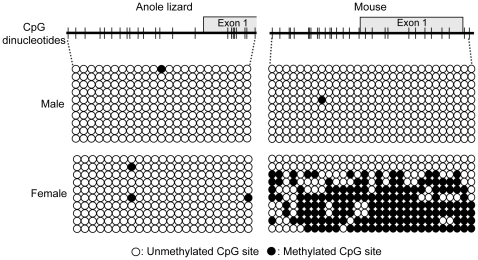
CpG methylation of the anole and mouse Ant2 genes. The CpG methylation of the *Ant2* gene promoter regions in female and male anole lizard and mouse was examined by bisulfite sequencing analysis. The schematic diagrams indicate CpG islands in the *Ant2* gene promoter regions examined in anole lizard and mouse. Each row of the circles represents an individual clone of a PCR amplicon in bisulfate sequencing analysis.

We previously demonstrated the promoter region of the murine *Ant2* gene is partially methylated at CpG dinucleotides in female somatic tissues, which is to the result of somatic X-chromosome inactivation of one allele in females. Here we examined CpG methylation in the promoter region of the *Ant2* gene in male and female tissue samples from anole lizards and mice. As we previously described, the murine *Ant2* promoter was unmethylated in male tail tissue, whereas female tail tissue showed hypermethylation of the *Ant2* promoter in approximately half of the samples examined (**Fig. 6**). In contrast, the anole *Ant2* promoter was largely unmethylated in both male and female liver samples. These data are consistent with the idea that *Ant2* is located on an autosome or undifferentiated regions of sex chromosomes in anole lizard.

## Discussion

Previously, we hypothesized *Ant4* emerged at least 150 million years ago [22] based upon gene conservation in both eutherian and metatherian mammals. Here we demonstrated that anole lizard has an authentic *Ant4* ortholog based upon a number of criteria, including phylogeny, intron-exon structure, synteny, and pattern of gene expression. Further, degenerate DNA fragments of a putative *Ant4* pseudogene were identified in the syntenic region of avian genomes. These indicate the presence of the Ant4 gene in the common amniote ancestor. This indicates that origin of the Ant4 gene clearly predates the divergence of mammals and reptiles, more than 300 MYA.

Phylogenetic analyses suggest that the gene duplication that led to the *Ant4* lineage and the *Ant1*, *2*, and *3* lineage may actually be much more ancient than the origin of amniotes. A number of invertebrate lineages were nested within the diversity of vertebrate *Ant* genes (**Fig. 2A**), a topology similar to the estimate of phylogeny obtained by Brower et al. [22]. However, there are a number of reasons that one can obtain an incorrect estimate of phylogeny [27], [28], and the branch length heterogeneity evident in the large-scale animal *Ant* tree is a major cause for concern (especially for the Brower [22] tree that was based upon NJ of protein distances that were corrected by the Kimura [34] formula). To address concerns regarding the phylogeny we used ML with the best fitting model (LG+Γ+F) to estimate the tree for this study, addressing the issue of model adequacy. We also examined two sets of sequences, one of which excluded the divergent outgroups and other relatively long branches (**Fig. 2B**) because divergent sequences can act as “rogues” that can distort the topology [29], [30], [35]. Rogue taxa are characterized with an unstable phylogenetic placement when relatively subtle details of analyses are changed and they can have a negative effect upon the estimation of both topology and bootstrap support values. Despite our concern regarding the inclusion of divergent, potentially rouge sequences, the estimate of phylogeny actually appeared quite robust to the specific sets of taxa that were included. However, the exclusion of divergent sequences did appear to improve the estimate of phylogeny in relatively subtle ways. Specifically, the topology for both the *Ant1* and *Ant3* subgroups were more congruent with the species tree (e.g., analyses of the large taxon sample placed the root within tetrapods whereas analyses of the smaller taxon sample placed it between fish and tetrapods, with the latter being more likely to be correct). Significantly, we found a higher degree of bootstrap support for the clade including vertebrate *Ant4* and tunicate *Ant4*-like sequences when the more limited taxon sample was analyzed, consistent with our hypothesis that the *Ant4* group is the result of an ancient duplication.

It is clear that all estimates of *Ant* phylogeny demand a number of duplications and losses to reconcile them with the animal tree of life. Some of the observed incongruence between expectation based upon the species tree and the estimates of *Ant* phylogeny may reflect the lack of power associated with relatively short protein sequences [36]. Indeed, fairly long sequences can be necessary to have a high probability of obtaining an accurate estimate of phylogeny [37], [38]. In addition to issues associated with the power of phylogenetic analyses to distinguish among alternative trees, the observed branch length heterogeneity (**Fig. 2**) suggests that the patterns of evolution for *Ant* genes have undergone substantial changes during animal evolution. More specifically, the branch length for the *Ant4* group is somewhat longer than those for the *Ant1*, *2*, *3* group, suggesting that the latter group has retained more of the ancestral functions of animal Ant proteins. Indeed the distance between the anole and human Ant4 (0.3608 amino acid substitutions per site, based upon patristic distances calculated from the tree in **Fig. 2B**) indicates that the rate of amino acid evolution was approximately 2.4-fold faster for the product of *Ant4* than it was for *Ant1*, *2*, and *3* gene products (the mean anole-human distance for the *Ant1-3* group was 0.1484 amino acid substitutions per site). These rate estimates based upon amino acid substitutions are consistent with estimates of ω (Table S2), emphasizing the fundamentally different patterns of sequence evolution for *Ant4* and *Ant1-3* groups.

The observed changes in the patterns of evolution for *Ant* genes may have impacted the estimate of *Ant* phylogeny, although the heterogeneity does not appear so extreme as to radically alter our conclusions regarding the evolution of the vertebrate *Ant* gene family. The basis for the higher rate of nonsynonymous sequence evolution evident in the *Ant4* group is unclear, although it is known that genes with sex-biased expression often exhibit higher rates of evolution [39]. The basis for the observed rate differences for sex-biased genes are complex [40], but it is important to note that our observation that *Ant4* exhibits a higher rate of evolution is consistent with a role for this gene in male reproduction in various amniotes. Alternatively, the higher rate of *Ant4* evolution may reflect changes in selection unrelated to sex-biased expression, either the relaxation of purifying selection, the action of positive selection having acted upon *Ant4*, or both phenomena. Indeed, when the branch length heterogeneity observed in our *Ant* trees (**Fig. 2**) is combined with the evidence for the action of natural selection upon other mitochondrial proteins [41], [42] it seems reasonable to speculate that various *Ant* proteins have been subject to episodes of positive selection during animal evolution. Regardless of the specifics, both hypotheses regarding *Ant4* (relaxed purifying selection or positive selection) are consistent with the idea that *Ant4* has undergone a shift in function.

Pseudogenes exhibit relaxed selection on nonsynoymous sites after the relaxation of purifying selection, and ω is expected to approach one after a period of time [43]. Although the fact that the ML estimates of ω for the birds were less than one (ω = 0.5146 for exons 1 and 2 and ω = 0.3921 for exon 2 alone; also see Table S1) might seem more consistent a relatively recent gene inactivation, it is important to note that the confidence interval on estimates ω after the relaxation of purifying selection tends to be quite wide [43]. Instead, the best-fitting models (Table S1) suggest the relaxation of purifying selection upon *Ant4* in birds occurred substantially earlier than the Passeriformes-Galliformes divergence at least 87 MYA [37]. It will be interesting to determine whether a functional *Ant4* gene is present in any other extant archosaurs (birds and crocodilians) and whether there are any phenotypic correlates of the *Ant4* gene loss.

The present study also urged us to reconsider our original hypothesis that was based upon the conservation of *Ant4* only in mammals, since it is clearly present both in mammals and anole lizards. Based on the unique chromosomal localization pattern of the *Ant* genes as described in the introduction, we originally hypothesized that the conservation of *Ant4* is driven by the sex chromosomal localization of the *Ant2* gene and the subsequent inactivation of *Ant2* during male meiosis in mammals. When we identified an authentic *Ant4* ortholog in anole lizard, we first expected the anole *Ant2* gene to be located on its sex chromosome as well, which would strengthen the “compensation” hypothesis. Among reptiles that have various sex determination systems, anoles are considered to have male heterogametic sex determination like mammals [32], [33]. Although the scenario was rather unlikely as sex chromosomal differentiation presumably developed independently in mammals and reptiles after the bifurcation of the two species, it is possible that the *Ant2* gene can be localized to sex chromosomes in both mammals and anoles by chance. Interestingly, an *Ant* gene homolog was isolated from heteromorphic sex chromosomes in the Japanese frog *Rana rugosa* [44], an unusual species where different populations have three distinct types of sex chromosomes (homomorphic XY chromosomes, heteromorphic XY chromosomes, and heteromorphic ZW chromosomes). Although the gene was originally annotated as *Ant3* in the report [44], it actually shows greater sequence identity to *Ant2* rather than *Ant3* (91% and 88% to human *Ant2* and *Ant3*, respectively). Placement of the *Rana rugosa* sequences using phylogenetic criteria was more equivocal, as they formed a clade with putative *Xenopus Ant3* ortholog (data not shown). However, the putative *Xenopus Ant3* sequence was placed either within fish (**Fig. 2**) or sister to fish (when the *Rana* sequence was added); this topology suggests either a local rearrangement in the tree (likely due to the relatively short length of the *Ant* sequences) or additional gene duplications and losses. The absence of bootstrap support for separating *Ant2* and *Ant3* (**Fig. 2**) is consistent with the first hypothesis, which postulates that the relevant branches in our estimate of phylogeny are rearranged relative to the actual evolutionary history. Regardless, it is clear that the sex chromosomal localization of a member of the *Ant2/3* group is not unique to mammals. There could be a yet unidentified common mechanism or reason why members of this subgroup of *Ant* genes have a higher probability of moving to a sex chromosome after their origin. However, it should be noted that *Ant2* gene localizes to autosomes in birds (chicken and zebra finch); thus the sex linkage of *Ant2* is not a universal phenomenon in vertebrates.

Chromosome maps are not completed to date for *Anolis carolinensis*, making it difficult to determine whether or not the anole *Ant2* gene is localized at a sex chromosome. Instead here we investigated relative gene dosage of the gene between females and males. In mice, dosage of the *Ant2* gene as well as its neighbor *Slc25a43* gene were approximately 2∶1 between females and males, consistent to the fact that these genes are localized at heterologous regions of the X chromosome. In contrast, in anoles, both *Ant2* and *Slc25a43* genes doses were 1∶1 between females and males. These data indicate that the *Ant2*-*Slc25a43* region is not likely on a heterologous region of the sex chromosomes. One of two X chromosomes in the somatic cells of female mammals is known to be inactivated accompanying CpG methylation. Indeed, we previously showed that the mouse *Ant2* gene was partially methylated in females but not in males [45]. Here, we demonstrated that anole *Ant2* gene was unmethylated both in females and males, which is consistent with the idea that the *Ant2* gene is not on the anole X chromosome. It should be noted, however, that there is no information available regarding the mechanism (or even the existence) of dosage compensation for sex-linked genes in anoles. Thus, it is unclear whether X chromosomes undergo methylation and/or inactivation in anoles in a similar mechanism as mammals, though it is known that reptilian genomes typically have a CpG methylation ratio comparable to that of other amniotes [46]. Although a definitive conclusion regarding the chromosomal location of *Ant2* in *Anolis carolinensis* will require an improved anole genome assembly, our present data suggest that the *Ant2* gene is not likely localized to a heterogametic sex chromosome in anoles.

Our results imply the conservation of *Ant4* may not be simply driven by the sex chromosomal localization of *Ant2* gene and its subsequent inactivation during male meiosis. Importantly, our data suggest that testicular expression of *Ant4* existed prior to and is independent of the sex chromosome linkage of the *Ant2* gene in mammals. This prompted us to hypothesize that Ant4 has been functioning originally in testicular germ cells and sperm, in the amniote ancestor and potentially even earlier in evolutionary phylogeny. The degeneration of *Ant4* in birds indicates that this *Ant* paralog is unnecessary for the development of testicular germ cells in this lineage. To this end, one alternative hypothesis could be that *Ant4* plays a critical additional role during spermatogenesis and/or in sperm function. This role would be shared by specific groups of organisms such as mammals and anoles (and probably by at least some other squamates) and absent in other groups of organisms including birds. Likewise, the absence of *Ant4* orthologs in the frog genome and all teleost fish genomes suggest it is absent from these lineages as well, and the existence of multiple fish genome sequences strongly suggests this absence is real rather than a problem with the completeness (or assembly and annotation) of the available draft genome sequences. Our phylogeny implies that duplication leading to the *Ant4* lineage predates the origin of vertebrates, indicating that the absence of *Ant4* in this lineage likely reflects gene loss. The loss of *Ant4* in birds and fish could reflect the relaxation of selection of this paralog or the existence of a specific advantage linked to the loss of this gene, since some evolutionary innovations can be linked to gene loss [47]. Regardless, it seems clear that conservation of *Ant4* in anoles and mammals cannot be explained by the simplest version of the compensation theory, which postulates selection to retain the *Ant4* gene after duplication reflects a need to compensate for the loss of *Ant2* expression during male meiosis in mammals. Instead it suggests that vertebrate *Ant4* orthologs may have additional specific functions associated with male germ cells prior to the need for compensation.

Then, what can be an additional ‘specific’ function of Ant4 that is essential for male germ cells only in anole and mammals? Interestingly, the *CatSper* genes are also conserved in mammals and the anole lizard, but not in birds, frogs, and bony fishes, similar to *Ant4* [48]. *CatSper* genes encode sperm-specific voltage-gated Ca^2+^ channels that are crucial for sperm hyperactivation and male fertility in mammals [49]. Hyperactivated motility in mammals occurs as the sperm encounter progressively more alkaline environments as they ascend the reproductive tract [50]. This sperm hyperactivation characterized by a large bend angle of head and tail is proposed to be required for sperm to penetrate the cumulus and thick wall of the zona pellucida in mammals [51]. It is proposed that the unique conservation pattern in species having *CatSper* genes reflect the necessity of sperm hyperactivation for fertility [48]. It has long been appreciated that phylogenetic profiles (patterns of gene presence and absence) can provide information regarding gene function [52]. Although this hypothesis remains speculative, the analogous patterns of gene conservation for *Ant4* and *Catsper* in vertebrates combined with the evidence that *Ant4* is expressed in sperm imply Ant4 might be critical for sperm hyperactivation. Since the hyperactivated motility likely requires increased ATP [53], it is reasonable to imagine that the ATP producing machinery, including the product of the *Ant4* gene, could be involved in hyperactivation. Although glycolytic ATP is largely implicated in mammalian sperm motility, it is still controversial whether mitochondrial ATP plays a role [54], [55]. As *Ant4* null mice exhibit meiotic arrest in male germ cells, we were unable to determine whether *Ant4* plays a specific role in mouse sperm after meiotic arrest. It would be interesting to examine whether expression of an *Ant2* transgene in *Ant4* null mice can fully compensate for the loss of *Ant4* or whether it is able to restore male germ cell meiosis but not in sperm hyperactivation. It should be noted, however, that multiple *CatSper* genes were lost in specific lineages, such as birds and fish, and that *CatSper* homologs are present in a number of invertebrates [48]. The tunicate *Ant4*-like genes appear likely to be orthologous to *Ant4* based upon phylogenetic criteria, but there is no other evidence (such as intron-exon structure) uniting them with vertebrate *Ant4* genes and it is unclear whether their function shows specific similarities to *Ant4*. Further evolutionary genomic analyses will be required to fully elucidate the similarities and differences among the Ant/Aac proteins expressed by distant species.

The idea that *Ant4* has an additional specific function in male gametes is supported by the fact that there are some differences in protein structure between Ant4 and the somatic Ant proteins [17], [18], [22]. There are common features seen only in Ant4, including N-terminal and C-terminal extensions and acidic amino acids between the end of the helix H5 region and the matrix M5 region. In addition, Dolce et al. [18] demonstrated that Ant4 has a lower affinity for adenine nucleotides than the other Ant proteins [56]. The structural and kinetic difference between somatic Ant proteins and Ant4 could imply that selection has optimized the Ant4 protein to function better in the helically arranged sperm mitochondria [57]. Alternatively, they could reflect the existence of a highly specific function associated with the Ant4 protein combined with relaxed selection of other biochemical properties. Interestingly, the ANT4 protein was isolated from the fibrous sheath of the human sperm flagellar principal piece using mass spectrometry proteomics and was shown to co-localize with glycolytic enzymes [19]. This study implied an additional function of Ant4 independent of mitochondrial oxidation. Although we were not able to detect Ant4 protein expression beyond the mid-piece of sperm in both mice and humans (unpublished data), in contrast to the report by Kim et al [19], the possibility that Ant4 protein has an extended localization and/or function is certainly an interesting hypothesis that needs to be clarified in the future.

## Materials and Methods

### Database mining, sequence alignment and phylogenetic analysis

Genomic sequences encoding Ant proteins from various species and their inferred amino acid sequences, including the anole lizard, were obtained from the Ensembl (http://www.emsembl.org), NCBI (http://www.ncbi.nlm.nih.gov), and JGI (http://genome.jgi-psf.org) databases (Appendix S1, S2, S3, S4, S5). Sequences were aligned using Mafft [58], [59] and imported into MacClade 4.08 (http://macclade.org) for manual adjustment. The maximum likelihood (ML) estimate of phylogeny was obtained using RAxML [60] version 7.2.8, using the LG model [61] with Γ-distributed rates across sites and empirical amino acid frequencies. We chose the LG+Γ+F model because it was the best-fitting model from the candidate set of empirical models. Model fit was assessed using the Akaike information criterion [62] using a neighbor-joining (NJ) tree estimated using uncorrected distances (*p*-distances). To assess support for specific groups in these we conducted 500 bootstrap replicates, using a full ML search for each replicate.

PAML 4.2 [63] was used to estimate the ratio of nonsynonymous substitutions per nonsynonymous site (*K*_A_) to synonymous substitutions per synonymous site (*K*_S_). The *K*_A_/*K*_S_ ratio (abbreviated ω) was estimated using alignments for each paralogous group (*Ant1*, *Ant2*, *Ant3*, and *Ant4*) that included anole lizard, human, mouse (*Mus musculus*), dog (*Canis familiaris*), and chicken (*Gallus gallus*) sequences. Nucleotide sequences were aligned and specific sequences that underwent gene loss (e.g., *Ant3* is not present in the mouse genome) were omitted. The topology was constrained to reflect the best current estimate of amniote phylogeny [27], [64]. A standard model of codon evolution with a single ω parameter for the entire tree was used for this analysis [65].

### Chromosome synteny

Syntenic chromosomal regions including *Ant4* gene among human, anole lizard, chicken, turkey (*Meleagris gallopavo*), and zebra finch (*Taenopygia guttata*) were examined using the comparative genomics information in the Ensembl and UCSC genome browser databases [66]. For the identification of degenerate DNA fragment of putative *Ant4* gene in chicken, the putative degenerated *Ant4* region of chicken was translated into an amino acid sequence and the amino acid sequence was compared to the protein encoded by the human *Ant4* gene. To determine when the shifts in the constrains on the avian *Ant4* orthologs occurred, we used PAML 4.2 [63] to estimate ω using a variety of models and an alignment that included anole lizard, human, mouse, and dog sequences in addition to the avian pseudogene sequences. The set of codon models examined to address this question included the standard single ω model [65] and branch models [67] with multiple free ω parameters (two parameters, three parameters, and a free ω parameter for all branches). Akaike weights [68], which can be interpreted as the probability that a specific model is the member of the candidate set with the smallest distance to the (unknown) true model, were calculated to compare the fit of the candidate models. The use of Akaike weights allowed both to rank models from best to worst and to define a 95% confidence set of models (the set of models that excludes those with low Akaike weights; the sum of the Akaike weights for the excluded models is ≤0.05 for the 95% confidence set).

### Animals and sample preparation

Anole lizards were rapidly decapitated, and tissue samples were immediately frozen on dry ice. They were stored at −80°C until use. For extraction of total RNA and genomic DNA, tissues were ground in liquid nitrogen using a mortar and pestle prechilled in liquid nitrogen. Murine tails were cut from 3 week old C57BL/6J when weaned and tails were stored at −20°C. All procedures performed on anole lizards and mice were reviewed and approved by the Institutional Animal Care and Use Committee (IACUC) of Michigan State University and the University of Florida.

### RNA expression (qRT-PCR)

Total RNA was isolated from heart, liver and testis of anole lizards using the RNAqueous kit (Ambion) according to the manufacturer's instructions and genomic DNA contamination was removed by the TURBO DNA-free™ kit (Ambion). Complementary cDNA was synthesized from total mRNA by reverse transcriptase using random primers (Applied Biosystems) under the following conditions; 25°C for 10 min, 37°C for 120 min, 85°C for 5 sec. The qRT-PCR was performed using the SYBR Green (Applied Biosystems) under the following thermocycler conditions; 95°C for 10 min and 40 cycles at 95°C for 15 sec and 60°C for 1 min. The levels of Ant mRNA in each tissue was normalized to the housekeeping gene β-actin and relatively quantified by the 2-ΔΔCt method. Template equivalent to 2.5 ng of total RNA was used for the amplification and each reaction was performed in triplicate. The primer sets used were: Ant1:5′-GACACGGTCCGACGTAGAAT and 5′ AAGAAAGCCTTGCCTCCTTC, Ant2:5′-CTACAGAGCGGCCTACTTCG and 5′-TCCAGCTGATCAAAATGTGG, Ant3:5′-TTGCGGGGTTTATATCAAGG and 5′- TCTGGGAGCATACCTTTTGC, Ant4:5′-AGACGCATGATGATGCAGAG and 5′-AAGGCTCCTCGAAAGAAAGC, and β-actin:5′-TCCCTGGAAAAGAGCTACGA and 5′-GCAGGACTCCATACCCAAAA.

### Gene dosage analysis (qPCR of genomic DNA)

The relative gene dosages of *Ant1*, *2*, *3* and *4* as well as *Slc25A43* (a neighboring gene to Ant2), between female and male genomes, were determined by qPCR of genomic DNA. Wizard Genomic DNA Purification Kit (Promega) was used to extract genomic DNA according to the manufacturer's protocols from livers of male and female anole lizards and tails of male and female mice as controls. A 2.5 ng sample of gDNA from each tissue was used as the template and qPCR was performed under the same thermocycler conditions as described above. Each Ant gene amplification was normalized to the β-actin of anole lizard and mouse, respectively and relative gene copy number was calculated by the 2-ΔΔCt method. Finally, the relative gene dosages in females and males were obtained by dividing the relative gene copy numbers in females by the relative gene copy numbers in males. The primer sets are listed below. Primer set I for anole lizard was Ant1:5′-ATGTCTCAGTCCAGGGCATC and 5′-TGCCACTTCCGAGACCTCTA, Ant2:5′-CTACAGAGCGGCCTACTTCG and 5′-AAACGGAAACACGGATACGA, Ant3:5′-CCTATCCCTTTGACACTGTGC and 5′-GCCATACAGCCCAGAAAACA, Ant4:5′-TTTTGGCTCCTTCAAAATGG and 5′-GATTTGGCAGCATTCCCTAA and β-actin:5′-CACTTGTGTTGCCTCACGTT and 5′-GGGGTGTTGAAGGTCTCAAA. Primer set II for anole lizard was Ant2:5′-AGTCTCTCTCCCGGTTCCTC and 5′-ATGGTGCCCGAGTACATGAT, Ant4:5′-GGGTGGAGGTTCTGCATCTA and 5′-CCGAAAGAGATGGGATCAAA, and β-actin:5′-CATTAGCCCTCGAACTTTGG and 5′-GGATACCGCAGGACTCCATA. Primer sets for mouse was Ant2:5′-TTCCCTTTCCCCTTCTCTGT and 5′-TATCTGCCGTGATTTGCTTG, Ant4:5′-GCGTCCTCCAAGCAGATAAG and 5′-TATGGGATGCTAAGGCCAAG and β-actin:5′-TGCCCTGAGTGTTTCTTGTG and 5′-GGGGTGTTGAAGGTCTCAAA.

### CpG methylation analysis (bisulfite sequencing)

The bisulfite conversion of genomic DNA extracted from tissues described above was carried out using EZ DNA Methylation-Gold™ Kit (Zymo Research). Putative Ant2 promoter regions in the bisulfite converted gDNAs of anole lizard and mouse were amplified by PCR with the following primer sets: Ant2 for anole lizard:5′-GTTGGATGTTTTATGAGGTTTTTTT and 5′-CCTAAAACAAAAACTTAATCCTCTC and Ant2 for mouse:5′-GGTTTGATTAGGTGTTAAGGGTAAG and 5′-AAAATACCCCCTTTCTATACAAATC. The amplicons were gel-purified and cloned into bacterial cells using TOPO TA cloning (Invitrogen). Ten colonies from each sample were chosen and sequenced.

## Supporting Information

Table S1 Fit of models of codon evolution to the *Ant4* nucleotide alignment.
(DOCX)

Table S2 Estimates of *K*_A_/*K*_S_ ratio (ω) for paralogous *ANT* groups.
(DOCX)

Appendix S1 Details of phylogenetic and evolutionary analyses.
(PDF)

Appendix S2 Details of phylogenetic and evolutionary analyses.
(NXS)

Appendix S3 Details of phylogenetic and evolutionary analyses.
(NXS)

Appendix S4 Details of phylogenetic and evolutionary analyses.
(TRE)

Appendix S5 Details of phylogenetic and evolutionary analyses.
(TRE)
