# Supplementary material for: Evolutionary Genomics Implies a Specific Function of Ant4 in Mammalian and Anole Lizard Male Germ Cells
Source: PLoS One. 2011 Aug 12;6(8):e23122. doi: 10.1371/journal.pone.0023122 (PMC3155547; doi:10.1371/journal.pone.0023122)
Supplement: Table S1 — Fit of models of codon evolution to the Ant4 nucleotide alignment. (DOCX) [file pone.0023122.s001.docx]

Table S1. Fit of models of codon evolution to the *Ant4* nucleotide alignment.

|  |  |  |  |  | Estimate of ω for: | | |
| --- | --- | --- | --- | --- | --- | --- | --- |
| Model *^a^* | Parameters*^b^* | *ln* L | AIC*^c^* | Akaike weight*^d^* | Within Birds | Branch Uniting Birds | Rest of Tree |
| Exon 2 alignment (includes Zebra Finch) | | | |  |  |  |  |
| Single ω | 13 | -454.0801 | 934.1603 | 0.0026 |  | ω = 0.1748 |  |
| Two ω, type 1 | 14 | -447.5573 | 923.1146 | 0.6475 | ω_1_ = 0.3921 | ω_1_ = 0.3921 | ω_0_ = 0.0477 |
| Two ω, type 2 | 14 | -449.4756 | 926.9513 | 0.0951 | ω_1_ = 0.4118 | ω_0_ = 0.0749 | ω_0_ = 0.0749 |
| Three ω*^e^* | 15 | -447.5372 | 925.0743 | 0.2430 | ω_1_ = 0.3804 | ω_2_ = 0.5116 | ω_0_ = 0.0468 |
| Free ω | 23 | -442.5595 | 931.1190 | 0.0118 | Different ω for each branch *^f^* | | |
| Exons 1 and 2 alignment (excludes Zebra Finch) | | | |  |  |  |  |
| Single ω | 11 | -714.4804 | 1450.9607 | 0.0033 |  | ω = 0.1835 |  |
| Two ω, type 1 | 12 | -708.5543 | 1441.1086 | 0.4602 | ω_1_ = 0.3921 | ω_1_ = 0.3921 | ω_0_ = 0.0477 |
| Two ω, type 2 | 12 | -709.6110 | 1443.2220 | 0.1600 | ω_1_ = 0.4118 | ω_0_ = 0.0749 | ω_0_ = 0.0749 |
| Three ω*^e^* | 13 | -707.9070 | 1441.8140 | 0.3235 | ω_1_ = 0.3804 | ω_2_ = 0.5116 | ω_0_ = 0.0468 |
| Free ω | 19 | -703.7162 | 1445.4325 | 0.0530 | Different ω for each branch *^f^* | | |

*^a^* Models are designated by the number of free ω (*K_A_/K_S_* ratio) parameters. The type 1 two-parameter model has one ratio (ω_1_) for the branches within birds and the branch uniting birds and a second (ω_0_) for the remainder of the tree whereas the type 2 model has one ratio (ω_1_) for the branches within birds and a second (ω_0_) for the remainder of the tree (including the branch uniting birds).

*^b^* Number of free parameters. Free parameters include the ω parameter(s), *κ* (transition/transversion ratio), and branch lengths.

*^c^* Akaike information criterion. The best-fitting model is indicated in red.

*^d^* Akaike weight. Models in the 95% confidence set are indicated in red.

*^e^* Note that the estimate of ω for the branch uniting birds (ω_2_) is actually higher than the estimate for birds (ω_1_). This is expected if the inactivation of *Ant4* was relatively early.

*^f^* Each branch is associated with a distinct ω value in this model. However, estimates of ω for the branches within birds and the branch uniting birds were high (e.g., for the branch uniting birds estimates of ω were 0.5845 [exon 2] and 0.3102 [exons 1 and 2]).
