# Supplementary material for: Evolutionary Genomics Implies a Specific Function of Ant4 in Mammalian and Anole Lizard Male Germ Cells
Source: PLoS One. 2011 Aug 12;6(8):e23122. doi: 10.1371/journal.pone.0023122 (PMC3155547; doi:10.1371/journal.pone.0023122)
Supplement: Table S2 — Estimates of K A/K S ratio (ω) for paralogous ANT groups. (DOCX) [file pone.0023122.s002.docx]

Table S2. Estimates of *K*_A_/*K*_S_ ratio (ω) for paralogous *ANT* groups

| Gene | ω |
| --- | --- |
| *Ant1* | 0.03805 |
| *Ant2* | 0.01747 |
| *Ant3* | 0.01930 |
| *Ant4* | 0.09910 |

PAML 4.2 [63] was used to estimate the ratio of nonsynonymous substitutions per nonsynonymous site (*K*_A_) to synonymous substitutions per synonymous site (*K*_S_). The *K*_A_/*K*_S_ ratio (abbreviated ω) was estimated using alignments for each paralogous group (*Ant1*, *Ant2*, *Ant3*, and *Ant4*) that included anole lizard, human, mouse, dog, and chicken sequences. Nucleotide sequences were aligned and specific sequences that underwent gene loss (e.g., *Ant3* is not present in the mouse genome) were omitted. The topology was constrained to reflect the best current estimate of amniote phylogeny [27,64]. A standard model of codon evolution with a single ω parameter for the entire tree was used for this analysis [65].
