# Supplementary material for: Evolutionary Genomics Implies a Specific Function of Ant4 in Mammalian and Anole Lizard Male Germ Cells
Source: PLoS One. 2011 Aug 12;6(8):e23122. doi: 10.1371/journal.pone.0023122 (PMC3155547; doi:10.1371/journal.pone.0023122)

Details of the phylogenetic and evolutionary analyses presented in **"Evolutionary Genomics Implies a Specific Function of *Ant4* in Mammalian and Anole Lizard Male Germ Cells"** by Lim et al.

Four nexus files are presented as supplementary information for Lim et al. These files provide the sequence alignments used for phylogenetic and evolutionary analyses as well as the relevant phylogenetic trees. The files are:

- **ANTalignment_annotated.nxs** – alignment of 94 protein sequences in nexus format. Analyses of this alignment (and a subset of 65 ingroup sequences) were conducted using RAxML 7.2.8.
- **ANT4_nuclaln_annotated.nxs** – alignment of 7 nucleotide sequences, including 4 full-length *Ant4* coding sequences and 3 partial *Ant4* pseudogenes sequences. This alignment includes the first 2 and last 3 nucleotides of the introns that are included in the pseudogene sequences.
- **ANTrooted.tre** – ML tree based upon of 94 ANT protein sequences, including the yeast outgroup.
- **ANTingroup.tre** – ML tree based upon of 65 ANT protein sequences, excluding the yeast outgroup and a number of divergent animal sequences.

These files are all in nexus format, allowing the commonly used phylogenetic analysis program PAUP* (http://paup.csit.fsu.edu) to be used for data management. Other programs that could also be used for data management, but examples of commands useful for data management are given only for PAUP*. Moreover, the files are written to echo instructions to the screen with proper formatting when executed in PAUP. Opening (or executing) the files in other programs may change details of the formatting. To make the information easier to read, all critical information is also presented in this file.

The tree files can be visualized in any program that reads nexus format (e.g., FigTree; http://tree.bio.ed.ac.uk/software/figtree) or loaded into PAUP* and visualized using the describetrees command. To make the information easier to read, the same information is presented in this file as supplementary figures.

Table of Contents:

- ANTalignment_annotated.nxs… page 2
- ANT4_nuclaln_annotated.nxs… page 4
- ANTrooted.tre… page 5
- ANTingroup.tre… page 6

Information about the annotated ANT protein sequence alignment

The annotation in the nexus file is presented below, with some edits and additional annotations (presented as italics) to improve readability in this format.

Alignment of ANT (mitochondrial adenine nucleotide translocase) proteins used in "Evolutionary Genomics Implies a Specific Function of *Ant4* in Mammalian and Anole Lizard Male Germ Cells" by Lim et al.

This alignment has been trimmed to remove the variable length sequences at the amino- and carboxyl-termini. When executed in PAUP* (http://paup.csit.fsu.edu) you can reconstruct the "ingroup" taxon set by executing the following commands before analyzing or exporting the data:

```
restore ingroup /only;
exclude ingroup_gaps;
```

(Many other nexus reader programs will allow similar manipulations of the data)

Note that some taxon names are longer than allowed for a standard phylip format file, so exporting in phylip format is not recommended. To convert to a "relaxed phylip" format (i.e., a format suitable for analysis in programs like RAxML simply edit this nexus file in a text editor or execute the following commands in a unix environment:

```
echo "94 309" > ANTalignment.phy
sed -n "/Matrix/,/;/p" ANTalignment_annotated.nxs | sed "1d" | sed "s/;//g" >> ANTalignment.raxml.txt
```

To produce the ingroup file substitute 'echo "65 301"' for the first command.

Sources of sequences:
Sequences from the Ensembl database are presented on the next page.

see below	*Saccharomyces cervisiae*, budding yeast outgroup	(NCBI)
	YMR056C, YBL030C, YBR085W	
MONBR	*Monosiga brevicollis*, choanoflagellate	(JGI)
NEMVE	*Nematostella vectensis*, starlet sea anemone	(JGI)
see below	*Caenorhabditis elegans*, nematode	(Ensembl)
	C47E12.2.2, W02D3.6, W02D3.6, K01H12.2, T01B11.4, F25B4.7, R07E3.4	
BRUMA	*Brugia malayi*, parasitic nematode	(NCBI)
PRIPA	*Pristionchus pacificus*, nematode	(Wormbase)
LOTGI	*Lottia gigantea*, gastropod	(JGI)
CAPTE	*Capitella teleta*, polychaete worm	(JGI)
HELRO	*Helobdella robusta*, leech	(JGI)
DAPPU	*Daphnia pulex*, water flea	(JGI)
see below	*Drosophila melanogaster*, fruit fly	(Ensembl)
	FBpp0073282, FBpp0073279	
STRPU	*Strongylocentrotus purpuratus*, sea urchin	(NCBI)
BRAFL	*Branchiostoma floridae*, Florida lancelet	(JGI)
OIKDI	*Oikopleura dioica*, appendicularian tunicate	(NCBI)

Information about the chordate sequences from the Ensembl database is presented below. The Ensembl (ENS) codes are also provided in this part of the table.

Tunicate1	*Ciona intestinalis*	ENSCIN	(Ensembl)
Tunicate2	*Ciona savignyi*	ENSCSAV	(Ensembl)
Anole	*Anolis carolinensis*	ENSACA	(Ensembl)
Cat	*Felis catus*	ENSFCA	(Ensembl)
Chicken	*Gallus gallus*	ENSGAL	(Ensembl)
Dog	*Canis familiaris*	ENSCAF	(Ensembl)
Frog	*Xenopus tropicalis*	ENSXET	(Ensembl)
Fugu	*Takifugu rubripes*	ENSTRU	(Ensembl)
Human	*Homo sapiens*	ENS	(Ensembl)
Medaka	*Oryzias latipes*	ENSORL	(Ensembl)
Mouse	*Mus musculus*	ENSMUS	(Ensembl)
Opossum	*Monodelphis domestica*	ENSMOD	(Ensembl)
Pufferfish	*Tetraodon nigroviridis*	ENSTNI	(Ensembl)
Rat	*Rattus norvegicus*	ENSRNO	(Ensembl)
Stickleback	*Gasterosteus aculeatus*	ENSGAC	(Ensembl)
Wallaby	*Macropus eugenii*	ENSMEU	(Ensembl)
Zebra_finch	*Taeniopygia guttata*	ENSTGU	(Ensembl)
Zebrafish	*Danio rerio*	ENSDAR	(Ensembl)

For the chordate sequences from Ensembl, the protein accession codes can be generated from the sequences below by replacing the taxon name with the ENS code shown above (e.g., the taxon name Anole_P00000011672 should be changed to ENSACAP00000011672)

Ensembl codes for human and anole genes are:

Gene	Human	Anole
ANT1	Human_P00000281456	Anole_P00000002268
ANT2	Human_P00000360671	Anole_P00000012600
ANT3	Human_P00000370808	Anole_P00000005895
ANT4	Human_P00000281154	Anole_P00000011672

As described above, these names can be converted to Ensembl protein codes by changing Human_ to ENS and Anole_ to ENSACA.

Note that the full and aligned sequences are presented in the text file.

Nexus format tree files for the ML analyses of this dataset (using the LG+G+F model) are also available. They can be visualized using programs such as FigTree (available from http://tree.bio.ed.ac.uk/software/figtree).

Information about the annotated *ANT4* nucleotide sequence alignment

The annotation in the nexus file is presented below, with some edits and additional annotations (presented as italics) to improve readability in this format.

Alignment of *ANT4* (*SLC25A31*) coding regions and avian *ANT4* pseudogenes used in "Evolutionary Genomics Implies a Specific Function of *Ant4* in Mammalian and Anole Lizard Male Germ Cells" by Lim et al.

This alignment includes the indels and other mutations in the pseudogenes as well as the ends of the intron sequences. This file includes several different charsets:

The intron sequences (only the ends of the introns are included):
 intron1 AND intron2
Alignments that include the pseudogenes (indels and nonsense codons excluded):
 aln_exons12 AND aln_exon2only
 Note that exon 1 is not present in the Zfinch sequence
Alignment that includes all coding sequences in the functional genes:
 all_coding

Codon positions are identified and they can be visualized using MacClade (http://www.macclade.org) or Mesquite (http://www.mesquiteproject.org).

To conduct analyses of the synonymous and non-synonymous rates in PAML (http://abacus.gene.ucl.ac.uk/software/paml.html) this file was executed in PAUP* (http://paup.csit.fsu.edu) and then the relevant regions were exported in phylip format. Since the aln_exons12 and aln_exon2only charsets exclude indels and nonsense codons, they can be analyzed using codon models in PAML.

Trees used for the PAML analyses (see Table S1 for results) were:

For exons 1 and 2:

1. tree for single omega and free omega
   `(Anole,(Chicken,Turkey),((Human,Dog),Mouse));`
2. tree for 2 omega, type 1 (early inactivation model)
   `(Anole,(Chicken #1, Turkey #1) #1,((Human,Dog),Mouse));`
3. tree for 2 omega, type 2 (late inactivation model)
   `(Anole,(Chicken #1, Turkey #1),((Human,Dog),Mouse));`
4. tree for 3 omega
   `(Anole,(Chicken #1, Turkey #1) #2,((Human,Dog),Mouse));`

For exons 2 only:

1. tree for single omega and free omega
   `(Anole,(Zfinch,(Chicken,Turkey)),((Human,Mouse),Dog));`
2. tree for 2 omega, type 1 (early inactivation model)
   `(Anole,(Zfinch #1,(Chicken #1,Turkey #1) #1) #1,((Human,Mouse),Dog));`
3. tree for 2 omega, type 2 (late inactivation model)
   `(Anole,(Zfinch #1,(Chicken #1,Turkey #1) #1),((Human,Mouse),Dog));`
4. tree for 3 omega
   `(Anole,(Zfinch #1,(Chicken #1,Turkey #1) #1) #2,((Human,Mouse),Dog));`

Information about the rooted ML tree estimated using the protein sequence alignment

The following tree was estimated in RAxML (http://wwwkramer.in.tum.de/exelixis) using the LG+Γ+F model and rooted to the yeast sequences that were included (YMR056C, YBL030C, YBR085W). See above for details about the data matrix.

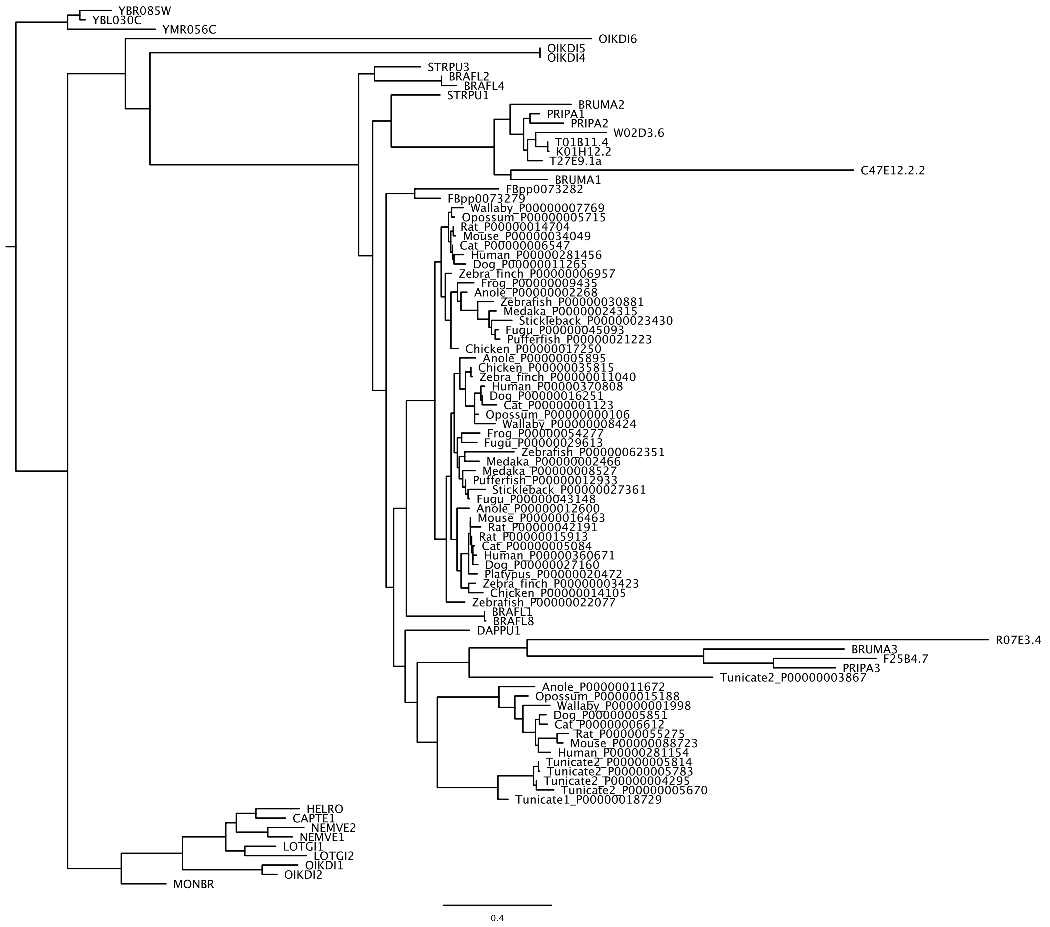

Information about the ingroup ML tree estimated using the protein sequence alignment

The following tree was estimated in RAxML (http://wwwkramer.in.tum.de/exelixis) using the LG+Γ+F. See above for details about the data matrix.

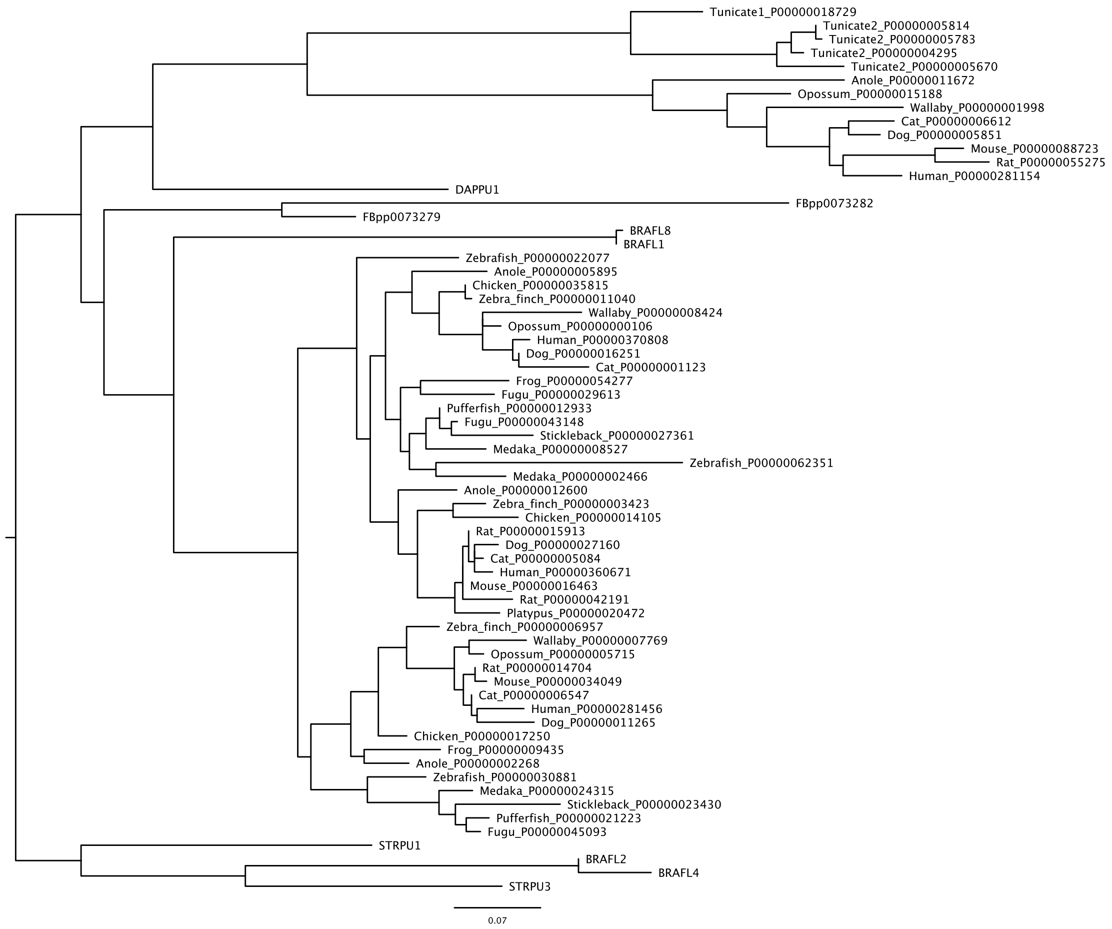

Supplement: Appendix S1 — Details of phylogenetic and evolutionary analyses. (PDF) [file pone.0023122.s003.pdf]
